# Antifibrinolytics in the treatment of traumatic brain injury

**DOI:** 10.1097/ACO.0000000000001171

**Published:** 2022-08-16

**Authors:** Patrick Schober, Stephan A. Loer, Lothar A. Schwarte

**Affiliations:** Amsterdam UMC, Vrije Universiteit Amsterdam, Department of Anesthesiology, Amsterdam, the Netherlands

**Keywords:** aminocaproic acid, antifibrinolytic, aprotinin, tranexamic acid, traumatic brain injury

## Abstract

Traumatic brain injury (TBI) is a leading cause of trauma-related deaths, and pharmacologic interventions to limit intracranial bleeding should improve outcomes. Tranexamic acid reduces mortality in injured patients with major systemic bleeding, but the effects of antifibrinolytic drugs on outcomes after TBI are less clear. We therefore summarize recent evidence to guide clinicians on when (not) to use antifibrinolytic drugs in TBI patients.

**Recent findings:**

Tranexamic acid is the only antifibrinolytic drug that has been studied in patients with TBI. Several recent studies failed to conclusively demonstrate a benefit on survival or neurologic outcome. A large trial with more than 12 000 patients found no significant effect of tranexamic acid on head-injury related death, all-cause mortality or disability across the overall study population, but observed benefit in patients with mild to moderate TBI. Observational evidence signals potential harm in patients with isolated severe TBI.

**Summary:**

Given that the effect of tranexamic acid likely depends on a variety of factors, it is unlikely that a ‘one size fits all’ approach of administering antifibrinolytics to all patients will be helpful. Tranexamic acid should be strongly considered in patients with mild to moderate TBI and should be avoided in isolated severe TBI.

## INTRODUCTION

Trauma is a leading cause of mortality, particularly in young adults, and accounts for about 4.4 million annual deaths worldwide [1]. Uncontrolled haemorrhage as well as traumatic brain injury (TBI) are the two leading causes of death after trauma [2], and in those patients with TBI, the presence and extent of intracranial bleeding is a strong predictor of mortality [3]. Hence, pharmacologic interventions to limit systemic as well as intracranial bleeding should have a large potential to reduce trauma-associated mortality, both in patients with extracranial injury and with TBI.

Antifibrinolytic agents have been shown to limit blood loss across a wide range of medical conditions, for example in patients with haemoptysis, epistaxis, haematuria or postpartum haemorrhage, as well as in patients undergoing major surgery, including cardiothoracic, abdominal, orthopaedic or obstetric surgery [4–13]. It therefore seems plausible that antifibrinolytics should also reduce blood loss in trauma patients. Since the publication of the landmark CRASH-2 trial on effects of tranexamic acid (TXA) in injured patients with (risk of) significant bleeding in 2010 [14], this drug has been widely used to prevent trauma-related death and has even been added to the WHO's list of essential medicines in 2011. However, the role of TXA or other antifibrinolytics to improve outcomes after TBI is less clear. Although these drugs should theoretically be beneficial as outlined above, potential harm – for example due to cerebral intravascular microthrombi, dural sinus thrombosis, other thromboembolic complications or promotion of seizure activity – is also plausible [15^▪^,^16^].

In the first section of our review, we briefly outline the process of fibrin formation, fibrinolysis and trauma-induced coagulopathy. Subsequently, the key pharmacologic properties of the clinically most relevant antifibrinolytic drugs, namely aprotinin, TXA and ε-aminocaproic acid (EACA) are described. In the final part, we discuss the role of antifibrinolytics in the treatment of TBI. 

**Box 1:**
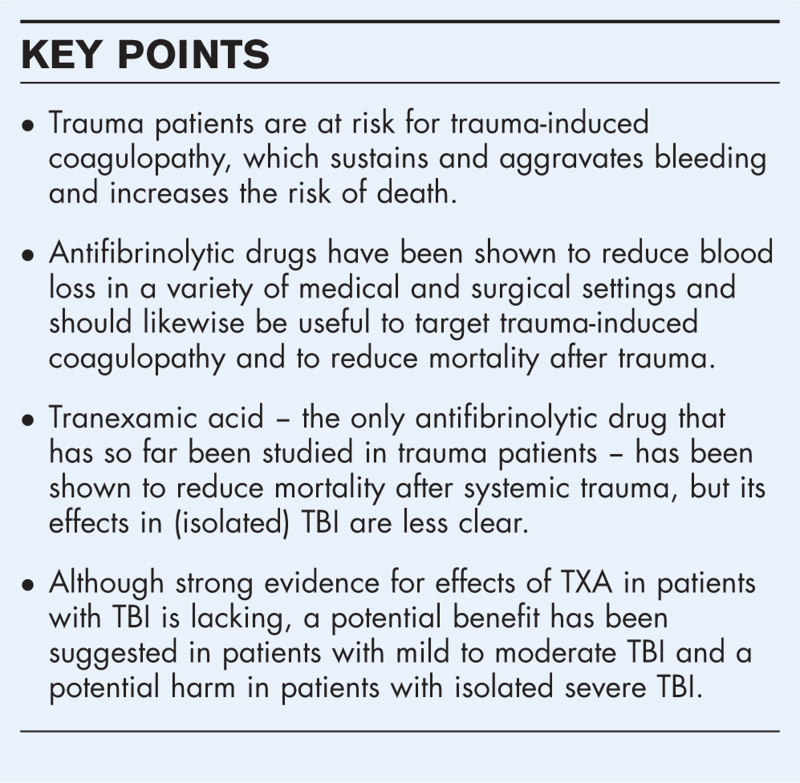
no caption available

## FIBRIN, FIBRINOLYSIS AND TRAUMA-INDUCED COAGULOPATHY

At the end of the coagulation cascade, fibrinogen (factor I) is converted to fibrin, which has a pivotal role in haemostasis, as fibrin polymers form a mesh that stabilizes the platelet clot and anchors it to the damaged blood vessel [17]. As a physiologic counterpart to clot formation, fibrinolysis serves to avoid excessive accumulation of intravascular fibrin and to prevent thrombosis, to dissolve existing thrombi and to degrade fibrin once the vascular damage has been repaired [18]. The key step in this process is the conversion of plasminogen to its active form, plasmin. This conversion is catalyzed by tissue plasminogen activator (tPA, main activator in blood), urokinase-type plasminogen activator (uPA) or other proteases [19,20]. Plasmin, in turn, cuts fibrin polymers into soluble elements known as fibrin degradation products, such as D-dimers.

Under physiologic conditions, coagulation as well as fibrinolysis are both tightly regulated by a number of activating or inhibiting enzymes or cofactors to maintain a balance between clot formation and clot dissolvement. However, under a variety of conditions such as (severe) surgical bleeding, cardiopulmonary bypass or trauma, the fragile equilibrium can be readily disturbed. In trauma patients, the bleeding that is initially caused by injury of blood vessels is often sustained and aggravated by an acute coagulopathy, referred to as trauma-induced coagulopathy (TIC) [21^▪^]. Although iatrogenic haemodilution by large volumes of crystalloids and other coagulation-factor free solutions as well as hypothermia and acidosis contribute to coagulopathy, TIC is a consequence of the trauma itself and occurs secondary to tissue injury, shock and inflammatory upregulation, independent of the aforementioned exogenous factors [22]. An early phase characterized by impaired coagulation and excessive bleeding (within 6 h after injury) is commonly distinguished from a late phase (onset >24 h after trauma) in which hypercoagulation prevails, but a large heterogeneity exists regarding the timing and clinical presentation of TIC [21^▪^]. The cause of TIC is multifactorial and involves endothelial activation, inflammatory upregulation, platelet dysfunction, impaired thrombin generation, fibrinogen depletion and hyperfibrinolysis, as reviewed in detail elsewhere [21^▪^,^22^]. Notably, coagulopathy and hyperfibrinolysis are not only common in patients with systemic injury and massive bleeding but are also regularly observed in patients with (isolated) TBI. This coagulopathy is commonly thought to be triggered by the release of tissue factor (factor III) from injured brain tissue [23] – which is actually an oversimplification given the complex interplay of various factors reviewed in detail elsewhere [24^▪^] – and its presence is associated with a markedly increased mortality [25].

In the context of traumatic bleeding and TIC, antifibrinolytic drugs are administered with the primary intention to limit blood loss by targeting (hyper-)fibrinolysis and shifting the balance towards clot stabilization. However, other properties of antifibrinolytic drugs, such as protective effects on the endothelium as well as anti-inflammatory effects, may also mediate beneficial effects in trauma patients [26–28]. Interestingly, given these properties, antifibrinolytics have also been increasingly used for indications completely unrelated to haemorrhage, for example for inflammatory skin disorders [29,30].

## PHARMACOLOGY OF ANTIFIBRINOLYTIC DRUGS

Two types of antifibrinolytics with different mechanism of action can be distinguished, namely serine protease inhibitors (aprotinin), as well as lysine analogues (TXA and EACA). Aprotinin is a nonspecific, competitive inhibitor that blocks the active sites of a family of enzymes known as serine proteases, which includes plasmin. Similar to the physiological plasmin inhibitor α_2_-antiplasmin, aprotinin primarily targets free plasmin but has little effect on bound plasmin [31,32]. Aprotinin is administered intravenously, and the typical dosing scheme for adult cardiac surgery, in which aprotinin has been predominantly used, consists of an initial loading dose of 2 million kallikrein inhibitor units (KIU) followed by a continuous infusion of 500 000 KIU per hour until chest closure, with an additional 2 million KIU added to the prime solution of the cardiopulmonary bypass circuit. Aprotinin is degraded by lysosomal enzymes and renally excreted, with a plasma elimination half-life of approximately 5–8 h [33].

After having been the most popular antifibrinolytic drug in the late 1990 s and early 2000 s, safety concerns raised in observational studies as well as in the *Blood Conservation Using Antifibrinolytics in a Randomized Trial* (BART) study [34] led to withdrawal of aprotinin from the market in November 2007. In the meantime, flaws identified in the BART-trial, as well as re-analyses of the available data have led to a re-evaluation of the risk-benefit profile, and aprotinin was reapproved in Canada in 2011 and in Europe in 2012, but is still unavailable in the United States.

**FIGURE 1:**
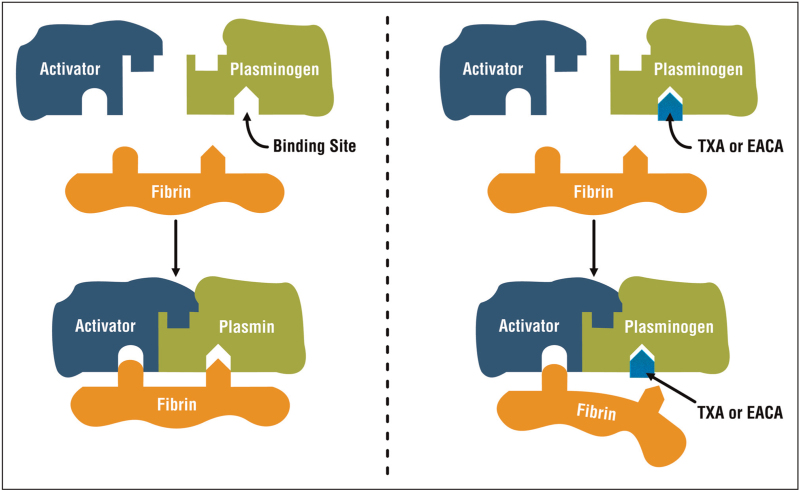
Mechanism of action of tranexamic acid and ε-aminocaproic acid. The left panel depicts binding of the activator (in blood, this is generally tissue plasminogen activator, tPA) and fibrin to plasminogen, leading to activation of plasminogen to plasmin and breakdown of fibrin into fibrin degradation products. TXA and EACA block the lysine binding sites of plasminogen (right), preventing the binding of fibrin.

Nowadays, EACA and particularly TXA are the most widely used antifibrinolytics. These synthetic analogues of the amino acid lysine competitively occupy the so-called lysine binding sites of plasminogen, which prevents binding of the fibrin molecule (Fig. 1) [35]. Both agents can be administered intravenously, orally and topically, and a recent clinical trial showed that TXA is also well tolerated and rapidly resorbed when administered via the intramuscular route [36,37]. In the context of anaesthesia, emergency medicine and critical care, TXA is usually administered to adults in an intravenous loading dose of 15 mg/kg or 1 g, followed by a continuous infusion of 1 g over 8 h. EACA is 6–10 times less potent than TXA [38] and therefore administered in higher doses, with a typical intravenous loading dose of 5 g given over 1 h, followed by a 1 g/h of continuous infusion for 8 h or until the bleeding is controlled. Both drugs are renally excreted unmetabolized, with a plasma half-life of about 2 h [39].

## USE OF ANTIFIBRINOLYTIC DRUGS IN TRAUMATIC BRAIN INJURY

Despite the theoretical potential of all antifibrinolytics to target TIC and to reduce bleeding in injured patients, no clinical data are available on effects of aprotinin or EACA in trauma patients. On the one hand, this can likely be explained by safety concerns about aprotinin and its continuing unavailability in the United States. On the other hand, due to the ubiquitous use of TXA in trauma patients following the CRASH-2 trial and given that TXA is a cheap and readily available drug, there has obviously been little interest to explore the usefulness of other antifibrinolytics for treating trauma-induced bleeding. Although it is not impossible that aprotinin and EACA may have a role in future clinical studies, all current evidence on the effects of antifibrinolytic drugs on outcomes in TBI is limited exclusively to TXA, on which the remainder of this section will focus. Table 1 summarizes the randomized trials specifically investigating the effects of TXA in patients with TBI, as well as recent observational studies (2019–2022).

**Table 1:** Randomized trials and recent observational studies investigating the effects of TXA in patients with TBI

Study	*n*	Population	Intervention	Outcome(s)	Key findings
Randomized studies					
Chakroun-Walha *et al.* (randomized open-label trial) [56]	180	Performed in Tunisia. Patients ≥ 15 years admitted within 24 h after TBI with intracranial bleeding on CT and no major extracranial bleeding were included when the responsible doctor was substantially uncertain as to whether to use TXA.	1 g TXA loading dose in 10 min followed by a maintenance dose of 1g for 8 h. Control group: no treatment with TXA.	Primary outcomes: reduction in the need for surgery or transfusion and mortality within 28 days after trauma. Secondary outcomes: Disability at 28 days after trauma and adverse events.	No significant difference in need for surgery or transfusion rates, no evidence for a difference in mortality, significantly higher rate of pulmonary embolism in TXA group.
CRASH-2 trial Intracranial Bleeding Study 2011 (double-blind RCT nested in the CRASH-2 trial) [41]	270	Performed in India and Colombia. Patients meeting CRASH-2 inclusion criteria (adult patients with (risk of) significant haemorrhage within 8 h of injury) with additional TBI (GCS ≤14 and a brain CT compatible with TBI).	1 g TXA loading dose in 10 min followed by a maintenance dose of 1 g for 8 h. Control group: placebo.	Primary outcome: total haemorrhage growth. Secondary and clinical outcomes include new intracranial haemorrhage, death from any cause at discharge or at 28 days, dependency, and the need for neurosurgical intervention.	No significant differences in haemorrhage growth or mortality dependency at hospital discharge or need for neurosurgical intervention.
CRASH-3 trial 2019 (double-blind RCT) [42^▪▪^]	9127	Performed in 29 countries, most patients included in Pakistan, UK and Malaysia. Adults within 3 h after injury with GCS ≤12 or intracranial bleeding on CT and no major extracranial bleeding.	1 g TXA loading dose in 10 min followed by a maintenance dose of 1 g for 8 h. Control group: placebo.	Primary outcome: head injury-related death in hospital within 28 days of injury. Secondary outcomes: early head injury-related death, all-cause and cause-specific mortality, disability, complications and adverse events within 28 days of randomization.	No significant difference in the risk of head injury-related death; sub-group analyses suggest beneficial effects of TXA on mortality in patients with mild-moderate TBI. No evidence for adverse events or complications. A sub-study (published separately) found no overall significant reduction of progressive or new haemorrhage.
Ebrahimi *et al.* (double-blind RCT) [57]	80	Performed in Iran, patients ≥ 18 years with TBI presenting at the Emergency Department within 8 h, with isolated subdural or epidural haemorrhage on CT and no major extracranial injury, and with need for neurosurgery.	1 g TXA loading dose in 10 min followed by a maintenance dose of 1 g for 8 h. Control group: placebo.	Volume of blood loss during and after surgery, haemoglobin, GCS after surgery, mortality.	Reduced blood loss with TXA, no significant difference in mortality.
Fakharian *et al.* (double-blind RCT) [58]	149	Performed in Iran, patients ≥ 15 years with isolated TBI or multiple trauma patients with TBI as the main problem, arrival at the hospital within 8 h of trauma, with nonpenetrating injury and any kind of traumatic intracranial bleedings without need for surgery during the first 8 h.	1 g TXA loading dose in 10 min followed by a maintenance dose of 1 g for 8 h. Control group: placebo.	Primary outcome: increase in the volume of intracranial bleeding; secondary outcomes: need for brain surgery, death, functional status based on the Glasgow outcome scale, new bleeding and mass effects.	No significant differences in the proportion of patients with an increase in the volume of intracranial bleeding, no significant differences in mortality or unfavourable neurologic outcome.
Jokar *et al.* (single-blind RCT) [59]	80	Performed in Iran. Patients ≥ 15 years within 2 h of injury, with acute intracranial haemorrhage <30 ml on CT scan and GCS ≥ 8 without need for surgery.	1 g TXA loading dose in 10 min followed by a maintenance dose of 1 g for 8 h. Control group: placebo.	Primary (and only) outcome: intracranial haemorrhage volume and volume expansion at 48 h.	Significantly attenuated volume expansion in TXA group.
Mojallal *et al.* (double-blind RCT) [60]	100	Performed in Iran. Patients > 18 years with traumatic cerebral haemorrhage within 8 h of injury, no craniotomy within 24 h.	1 g TXA in 1 h. Control group: placebo.	Volume and progression of intracranial haemorrhage; death within 7 days; ICU length of stay.	No significant difference in mortality or volume of haemorrhage; shorter length of ICU-stay in TXA.
Mousavinejad *et al.* (double-blind RCT) [61]	40	Performed in Iran. Patients > 18 years within 8 h of injury, with brain contusion and intraparenchymal haemorrhage in CT scan, need for surgery, no significant extradural bleeding,	1 g TXA loading dose in 10 min followed by a maintenance dose of 1 g for 8 h [authors write 8 min but likely mean hours]. Control group: placebo.	Volume of blood loss during surgery, haemoglobin levels, mortality.	No significant differences in surgical blood loss, haemoglobin or mortality.
Rowell *et al.* (double-blind RCT) [54^▪▪^]	966	Performed in the United States and Canada. Patients ≥ 15 years with a prehospital GCS 3–12, at least one reactive pupil and systolic blood pressure ≥90 mmHg.	Either 1 g TXA as prehospital loading dose followed by 1g for 8 h after hospital admission or 2 g TXA as prehospital loading dose. Control group: placebo.	Primary outcome: functional neurologic outcome 6 months after injury. Secondary outcomes include 28-day mortality, progression of intracranial haemorrhage and adverse events.	No significant difference in functional neurologic outcome, 28-day mortality, progression of intracranial haemorrhage.
Safari *et al.* (double-blind RCT) [62]	94	Performed in Iran. Patients aged 15–65 years with traumatic intracerebral haemorrhage in initial CT without need for surgery and with GCS >3.	1 g TXA within 3 h of admission, followed by 1 g every 6 h for 48 h. Control group: placebo.	Haematoma expansion and level of consciousness.	Significantly reduced increase in haematoma volume in TXA group; no significant differences in level of consciousness at discharge.
Yutthakasemsunt *et al.* (double-blind RCT) [63]	238	Performed in Thailand. Patients >16 years with GCS 4–12 and CT within 8 h and without immediate indication for surgery.	2 g TXA as a single dose (as reported in the abstract) or 1 g TXA loading dose in 30 min followed by a maintenance dose of 1 g for 8 h (as reported in the main text). Control group: placebo.	Primary outcome: Haematoma expansion by 25% or more. Secondary outcomes included death and functional status at hospital discharge.	No significant differences in haematoma expansion, mortality or unfavorable outcome.
Recent observational studies published in 2019–2022					
Chan *et al.*[69]	651	Performed in Hong-Kong. Patients ≥ 18 years with an ICD code of ‘Cerebral contusion (852.19)’ or ‘Traumatic subarachnoid haemorrhage (852.00)’ as the primary diagnosis.	1 g TXA loading dose followed by 500 mg every 6 h for 24 h at the discretion of the treating physician.	Primary outcome: mortality within 30 days. Secondary outcomes include thromboembolic complication rates.	In the multivariable model, no significant relationship between TXA and mortality was found. Thromboembolic complication rates were not significantly different but do not seem to have been analyzed with multivariable models.
Morte *et al.*[70]	92	Individuals injured in combat setting in Iraq and Afghanistan.	Per protocol 1 g within 3 h of injury followed by 1 g for 8 h according to Combat Casualty Care Data guidelines.	Primary outcomes: GCS at discharge and in-hospital mortality. Secondary outcomes included rates of respiratory failure and thromboembolic events.	TXA was associated with better GCS at discharge and lower mortality.
Yap *et al.*[71]	334	Performed in Malaysia. Patients ≥ 12 years and above with isolated mild to severe TBI with any intracranial bleeding in CT brain within 8 h of injury, excluding GCS 3 with fixed and dilated pupils or CPR.	1 g TXA with 1 g infusion over 8 h at the discretion of the treating physician.	Primary outcomes: extended GOS ≥5 at discharge or 30 days (whichever was first). Secondary outcomes included expansion of intracranial blood volume, adverse events and death.	No significant differences in functional outcome or mortality was observed, but expansion of intracranial blood was lower in TXA treated patients.
Bossers *et al.*[15^▪^]	1827	Performed in the Netherlands. Patients with GCS ≤ 8 and injuries or a trauma mechanism suggestive for TBI, excluding CPR.	Prehospital administration of TXA (dose at the discretion of treating physician, usually 1 g).	Primary outcome: mortality within 30 days. Secondary outcomes: mortality at 1 year, functional outcome at discharge and 1 year.	No overall relationship between TXA and mortality after adjustment for confounders; subgroup analyses suggest an increased mortality after TXA in patients with isolated severe TBI.
van Wessem *et al.*[72]	234	Performed in the Netherlands. Polytrauma patients with Abbreviated Injury Scale - head ≥3 admitted to the adult ICU.	1 g TXA with 1 g infusion over 8 h at the discretion of the treating physician.	Primary outcome: hospital mortality. Secondary outcome: complications and adverse events.	No significant difference in mortality or complications.

The CRASH-2 trial was a large-scale study with 200 127 patients in 274 hospitals in 40 countries [14]. Adult trauma patients with significant bleeding, or at risk for significant bleeding, were randomized to receive either 1 g of intravenous TXA over 10 min followed by an infusion of 1 g over 8 h or placebo. The primary outcome was hospital mortality within 28 days of injury. The authors observed an absolute 1.5% lower mortality in patients treated with TXA (14.5 vs. 16%, *P* = 0.0035). Despite concerns about the external validity and other limitations summarized by Napolitano *et al.*[40], and despite the fact that TXA is not specifically approved for this indication by major drug agencies including the U.S. Food and Drug Administration (FDA) and the European Medicines Agency (EMA), TXA has since then ubiquitously been used (off-label) in trauma care worldwide. A nested substudy (CRASH-2 Intracranial Bleeding Study, [41]) provided first randomized data on the effects of TXA in patients with TBI [Glasgow Coma Scale (GCS) score ≤14 and a brain computed tomographic (CT) scan compatible with TBI]. With a sample size of only 270 patients, no significant differences were observed in the primary outcome (haemorrhage growth), or in individual clinical outcomes including death. Only when pooling several adverse outcomes into a ‘composite poor outcome’, a benefit of TXA was observed.

The CRASH-3 trial, published in 2019, is the first major and up to now the largest clinical trial of the effects of TXA in patients with TBI [42^▪▪^]. The study was performed in 175 hospitals in 29 countries. A total of 12 737 adult patients with a GCS score of 12 or less and any intracranial bleeding on CT scan and no major extracranial bleeding were randomized to receive either TXA or placebo with the same dosing scheme as in the CRASH-2 trial. Although the study initially included patients within 8 h of injury, the protocol was amended during the study period to limit inclusion to patients within 3 h of injury. This change reflected accumulating evidence that TXA should be administered as early as possible, and it has been suggested that administration after 3 h even increases mortality [43]. The amended primary outcome, death from head-injury within 28 days in patients randomized within 3 h of trauma, was analysed in 9127 patients, and no significant difference was found [18.5% in the TXA group vs. 19.8% in the placebo group, relative risk 0.94 (95% confidence interval, 95% CI 0.86–1.02)]. Similarly, there was neither evidence of benefit from TXA on key secondary outcomes, including all-cause mortality and disability, nor of an increase in adverse events and complications. A subgroup analysis suggested a protective effect of TXA in patients with mild to moderate TBI (GCS ≥ 9), but not in patients with severe TBI (GCS ≤ 8).

Despite actually being a ‘negative’ study with respect to the primary outcome, the authors of the CRASH-3 trial concluded that treatment with TXA within 3 h reduces head-injury related death. Although it is possible that this is true (a nonsignificant difference does not exclude a beneficial effect), the data actually do not provide strong evidence to claim benefit of TXA, particularly not in the overall cohort as well as in patients with severe TBI. The conclusion may be justified for patients with mild to moderate TBI, but the result found in this subgroup should also be interpreted with caution, as it carries an increased risk for type I error in the absence of a multiplicity adjustment [44]. Moreover, while CRASH-3 was a large and well designed trial, the study results must be viewed in the context of its limitations, such as concerns about the external validity of the results and potential selection bias [45–53].

Rowell *et al.*[54^▪▪^] performed another landmark-trial in which the effect of prehospitally administered TXA was studied. Patients at least 15 years of age with a prehospital GCS 3–12, at least one reactive pupil and a systolic blood pressure of at least 90 mmHg were randomly assigned to two different dosing schemes of TXA (1 g bolus and 1 g maintenance dose or 2 g bolus without maintenance dose) or placebo. Although the researchers initially planned to compare each TXA dosing group to placebo, concerns about the study power led to a protocol change and the two TXA groups were combined for comparison with the placebo group. The primary outcome was the extended Glasgow Outcome Scale score (GOSE) at 6 months after injury, dichotomized as a favourable (GOSE >4) or an unfavourable (GOSE ≤4) outcome. Of the 1063 study participants, 966 were analysed. There was neither a significant difference between the groups for the primary outcome nor for key secondary outcomes, including 28-day mortality, 6-month Disability Rating Scale score and progression of intracranial haemorrhage.

In a similar study of prehospital TXA administration [55], trauma patients at risk for haemorrhage were randomized to receive one of three different TXA treatment regimens or placebo, and the TXA groups were pooled for comparison with placebo. No benefit of TXA was observed for the primary outcome, 30-day mortality or for secondary outcomes. Although this study was not specifically designed to address TBI, no benefit was also observed in the subgroup of patients with TBI (*n* = 168).

Several smaller randomized trials also failed to show beneficial effects of TXA in terms of survival or neurologic outcome [56–63]. Five recent meta-analyses pooled the available evidence across published randomized trials, of which three reported beneficial effects of TXA [64–66] and two found no benefit [67,68^▪▪^]. Remarkably, all three studies that report beneficial effects inappropriately included the CRASH-2 trial – not only the patients with TBI from the intracranial bleeding sub-study but also all patients – so that the positive findings are largely attributable to CRASH-2 and do not specifically apply to the population of TBI patients. Hence, the available pooled evidence does not demonstrate a clear benefit of TXA in patients with TBI.

Recent observational studies have shown mixed results, including benefit as well as harm, as summarized in Table 1[15^▪^,^69^–^72^]. Although these studies must be interpreted with caution given their inherent limitations, in particular confounding [73], they allow gauging potential treatment effects of TXA when used in regular clinical practice rather than under controlled trial conditions. Notably, in an analysis of 1827 patients with severe TBI (GCS ≤8), Bossers *et al.*[15^▪^] did not observe an overall relationship between TXA and mortality after thorough adjustment for potential confounders, but found an increased mortality in TXA-treated patients in the subgroup of patients with isolated severe TBI.

**FIGURE 2:**
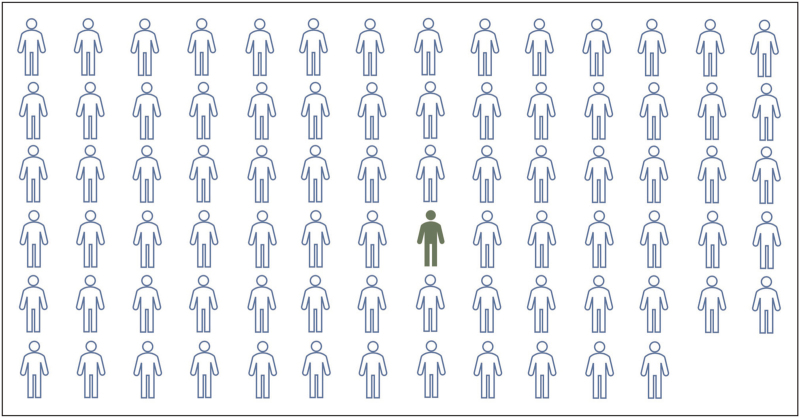
Visual impression of the effect size of tranexamic acid observed for the overall population of patients in the CRASH-3 trial. Assuming that the observed point estimate represents the ‘true’ effect of tranexamic acid in the population rather than random sampling error, 82 patients need to be treated to avert a single head-injury related death (solid pictogram), that is the vast majority receiving the drug do not benefit (outlined pictograms).

Considering all available studies jointly, the evidence for using TXA in TBI patients is rather weak. There is no clear evidence of either benefit or harm, and reported effect sizes were generally small. For example, assuming that the point estimate represents a true population effect rather than random sampling error, the overall effect seen in the CRASH-3 trial corresponds to a number needed to treat of 82 patients, that is a large number of patients need to be treated to avert a single death (Fig. 2). TXA is obviously not a magic bullet, and its use should not distract healthcare providers from focusing on those factors that are known to really matter for patient outcome, such as maintaining an adequate blood pressure [74]. Given the various factors that may influence the effect of TXA, such as the timing of administration, type of injury (isolated TBI vs. combined with extracranial haemorrhage) and severity of injury, as well as additional factors such as treatment infrastructure (e.g. access and transport times to definite care), it is unlikely that a ‘one size fits all’ approach of treating all patients with TXA will be helpful. The question arises as to which patients, if any, may actually benefit from this treatment. Although further studies are needed to provide additional insight, our personal interpretation and treatment recommendation based on the available literature is as follows:

(1) In patients with TBI and additional relevant extracranial bleeding, administration of TXA should be strongly considered on the basis of CRASH-2 and other studies demonstrating a lower risk of death in bleeding trauma patients.
(2) In patients with isolated mild to moderate TBI (GCS ≥ 9), CRASH-3 suggests a beneficial effect of TXA without an evident increase in adverse events. Thus, TXA is potentially lifesaving and should be strongly considered.
(3) For patients with isolated severe TBI, there is no evidence from randomized trials that TXA is beneficial, and observational data signal potential harms. TXA should therefore be avoided.
(4) Whenever TXA is being considered, it should be administered as early as possible and no later than 3 h after the injury. However, this treatment should not delay prompt treatment of factors known to trigger secondary brain injury.

## CONCLUSION

Although TXA appears to improve outcomes in injured patients with (or at risk for) major bleeding, the effects in patients with (isolated) TBI are less clear. Unequivocal evidence for benefit across all patients with TBI is lacking, and further research is needed to determine which patients actually do profit from TXA administration. Current literature suggests that TXA may decrease mortality in patients with mild to moderate TBI but may increase mortality in patients with isolated severe TBI.

## Acknowledgements

*The authors thank Daniel Rennen for designing and providing* *Fig. 1* *free of charge.*

### Financial support and sponsorship

*None*.

### Conflicts of interest

*There are no conflicts of interest.*

## REFERENCES AND RECOMMENDED READING

Papers of particular interest, published within the annual period of review, have been highlighted as:

▪ of special interest
▪▪ of outstanding interest
